# Supplementary material for: “I became a person again”: Social inclusion and participation experiences of Ethiopian women post-obstetric fistula surgical repair
Source: PLoS One. 2024 Jul 11;19(7):e0307021. doi: 10.1371/journal.pone.0307021 (PMC11238981; doi:10.1371/journal.pone.0307021)
Supplement: Attachment — Submitted filename: Response to reviewers.docx [file pone.0307021.s001.docx]

**Dear Sidrah Nausheen**

**Academic Editor for Plose one**

Thank you to all the reviewers who provided valuable feedback regarding the article submission titled “I Became a Person Again”: Social Inclusion and Participation Experiences of Ethiopian Women post-Obstetric Fistula Surgical Repair (Reference no. PONE-D-23-41017). We have addressed all the comments and questions and revised the manuscript accordingly. All the changes are tracked in the uploaded revised manuscript. As requested, we have also uploaded the clean copy. Please see below for our specific responses to each of reviewers’ points.

| **S.N** | **Reviewers comments** | **Response** |
| --- | --- | --- |
| 1. | The manuscript is excessively lengthy (50 pages), which diverts readers' attention from the key messages. Adjusting the word count and reducing repetition of concepts and content would improve readability. Additionally, the format should align more closely with that of an article rather than a thesis. | We reduced the length of the manuscript by ten pages, focusing on eliminating repetition, enhancing readability, and adhering to the article format. In the process, we also condensed and removed several sections, including some reference lists that were cited. |
| 2. | The content of the tables shows duplication of the term "vesicovaginal fistula," which appears to be a typo error. Consistency in the number of digits after the decimal point should be ensured throughout, and percentages should sum up to 100 for the presented data. | We appreciate you noticing this omission. The typo error has been corrected, and the duplicated term has been removed. All the percentages in the table sum up to 100%. Please refer to page 7. |
| 3. | The document requires a thorough review for minor language flow and grammatical errors. | We thoroughly reviewed and edited the manuscript for flow and grammar. |
| 4. | Details are missing regarding how privacy was ensured during interviews conducted in various locations, such as churches and homes. | This information is now provided on page 8, line 149. We added, “To ensure privacy, when conducting the interviews at home, we arranged for interviews to be in separate rooms and rescheduled if others were in the household. When interviews took place at a church or public institution, we selected weekdays when the location was empty or contacted the administrator to secure a private location.” |
| 5. | Additionally, information on the data collection process, interview timings, and participant selection criteria would enhance transparency and reproducibility. | Thank you for this comment. The data collection, interview process, and participant selection criteria are available in the main document. Please refer to pages 7-9. Also, we have added information regarding the timing of the interviews on page 8, line 145, as follows: “All interviews were completed in person; 16 were conducted in the morning, and the remaining five in the afternoon.” |
| 6. | What measures were taken to ensure reflexivity and transparency regarding the researcher's biases and assumptions should be elaborated upon. | Thank you for this comment. We have added information on reflexivity on page 9, line 168:  “Ahead of the study, the first author wrote the reflexivity statement examining the various facets of identity and experiences that could influence the analysis and interpretation of the results. The first author kept the reflexive journal throughout the study and discussed reflections with the last author (PhD supervisor) and the research team, as appropriate.” |
| 7. | Evidence of data saturation should be provided to ensure completeness of data collection. | The following was added on page 7, line 131:  “Data collection and analysis were undertaken simultaneously through an iterative process following the grounded theory approach of Charmaz (Charmaz, 2006). The data started to repeat at the 19^th^ participant, and we continued until the 21^st^ participant when the research team agreed that saturation was achieved as no new concept had emerged (Aldiabat, 2018).” |
| 8. | Differences in experiences and inclusiveness based on factors such as the type of fistula, duration since surgery, and the extent of incontinence or number of surgeries is lacking. | The following was added regarding the type of fistula, duration since surgery, and the extent of incontinence on page 7, line 135.  “Most participants had vesicovaginal fistula. More than half of the study participants had experienced stillbirth. Two-thirds of the participants were fully continent at the time of the interview. Table 1 illustrates the details of the study participants.” |
| 9. | The study's transferability aspect is commendable, with rich descriptions of findings. however, a discussion of the study's limitations and constraints affecting transferability needs consideration. | We have added the following two sentences on page 35, line 703, to clarify this limitation:  “The study findings represent the views of 21 women from Ethiopia and may have limited transferability beyond the specific context of the study.” |
| 10. | In the conclusion section, comments on the implications of such an extensive review and potential policy changes are missing. | We have added the statement about the potential policy implications on page number 36 line 714: “ The findings of this study have potential policy and service implications as they highlight the significance of a comprehensive approach to obstetric fistula care that extends beyond surgical measures and institution-based service to more continuous community-based programs focusing on addressing women's sexual and psychological health, and social participation.” |

Thank you for your consideration. We look forward to hearing back from you.

**Tibeb Debele, PhD Candidate in Rehabilitation Science**

**School of Rehabilitation Therapy, Queens University, Canada**

**Tibeb Debele**
