# Supplementary material for: “I became a person again”: Social inclusion and participation experiences of Ethiopian women post-obstetric fistula surgical repair
Source: PLoS One. 2024 Jul 11;19(7):e0307021. doi: 10.1371/journal.pone.0307021 (PMC11238981; doi:10.1371/journal.pone.0307021)
Supplement: Attachment — Submitted filename: Responce to reviewers.docx [file pone.0307021.s002.docx]

**2024/06/21**

**Dear Sidrah Nausheen**

**Academic Editor for PLOS ONE**

We want to express our gratitude to all the reviewers for their insightful feedback on the article submission titled “I Became a Person Again”: Social Inclusion and Participation Experiences of Ethiopian Women post-Obstetric Fistula Surgical Repair (Reference no. PONE-D-23-41017). In response to your request, we have successfully reduced the word count of our manuscript to around 7000 words, which is nearly 1100 words less than the second review. Considering the uncertainty regarding the exact word limit for the journal, we endeavored to find recently published articles with word counts ranging from 7000 to 8300, and we believe that our revised word count aligns closely with these parameters. We trust this meets your requirements and aligns with the journal's guidelines.

We appreciate your consideration and look forward to hearing back from you.

**Tibeb Debele, PhD Candidate in Rehabilitation Science**

**School of Rehabilitation Therapy, Queens University, Canada**

**Tibeb Debele**
